# Increased Risk to Develop Hypertension and Carotid Plaques in Patients with Long-Lasting *Helicobacter pylori* Gastritis

**DOI:** 10.3390/jcm11092282

**Published:** 2022-04-19

**Authors:** Maria Pina Dore, Pier Sergio Saba, Giulia Tomassini, Caterina Niolu, Marco Monaco, Giovanni Mario Pes

**Affiliations:** 1 Dipartimento di Scienze Mediche, Chirurgiche e Sperimentali, University of Sassari, Viale San Pietro 8, 07100 Sassari, Italy; giuliatomassini30@gmail.com (G.T.); caterina.niolu@hotmail.com (C.N.); monacomarco13@hotmail.it (M.M.); gmpes@uniss.it (G.M.P.); 2 Department of Medicine, Baylor College of Medicine, One Baylor Plaza, Houston, TX 77030, USA; 3 Clinical and Interventional Cardiology, Sassari University Hospital, 07100 Sassari, Italy; piersergio.saba@aousassari.it; 4 Sardinia Longevity Blue Zone Observatory, 08040 Ogliastra, Italy

**Keywords:** hypertension, *Helicobacter pylori* infection, Sardinia

## Abstract

*Helicobacter pylori* infection has been reported to be positively associated with hypertension, although with conflicting results. In this study, the relationship between *H. pylori* infection and hypertension, as well as atherosclerotic carotid lesions, was analyzed. Methods. Clinical records of patients referred to undergo upper endoscopy and gastric biopsy were retrieved. Information regarding the presence of *H. pylori* infection with atrophy/metaplasia/dysplasia (interpreted as a long-lasting infection), and current or past *H. pylori* infection was collected, as well as demographic variables, smoking habits, body mass index (BMI), dyslipidemia, diabetes, hypertension, presence of carotid lesions, and current treatment, and analyzed by multivariable regression models. Results. A total of 7152 clinical records from patients older than 30 years (63.4% women) were available for the study. Hypertension was present in 2039 (28.5%) patients and the risk was significantly increased in those with long-lasting *H. pylori* infection after adjusting for age decades, sex, BMI, cigarette smoking, diabetes, and dyslipidemia (OR 1.17, 95% CI 1.02–1.35). In addition, the long-lasting *H. pylori* infection was an independent risk for carotid plaques (OR 2.15, 95% CI 1.14–4.09). Conclusions. Our retrospective study demonstrated that long-lasting *H. pylori* infection is an independent risk factor for hypertension and the presence of carotid lesions after adjusting for potential confounders, although further validation our findings is needed from prospective studies.

## 1. Introduction

The incidence of cardiovascular disease (CVD) is increasing worldwide and represents a serious concern both in affluent and underdeveloped countries [1]. The number of deaths from CVD in 2020 was estimated to be 17.9 million, equivalent to 32% of all deaths globally [2]. The basic pathogenic process underlying CVD is atherosclerosis, a mechanism promoted by hypertension and other known risk factors such as age, male sex, cigarette smoking, obesity, dyslipidemia, diabetes, salt intake, and familial propensity [3]. Apart from these conventional risk factors, new potential cardiovascular risk factors are increasingly recognized [4], including infectious diseases [5,6,7,8,9,10]. Among microorganisms, *Helicobacter pylori* (*H. pylori*) is one of the most studied pathogens [11]. *Helicobacter pylori* is still responsible for widespread chronic bacterial infections [12,13], and its relevance promoting atheroma development is believed to be mediated by a chronic low-grade inflammation, triggered by the release of pro-inflammatory molecules, increased fibrinogen, C reactive protein, triglycerides, and low-density lipoprotein, which participate in the atherosclerotic process and promote a prothrombotic state [11,14,15,16].

Since 1988, Sonnenberg has suggested that gastric diseases and hypertension-related organ damage may share a common etiologic factor based on the results of an epidemiological study [17]. Similarly, an old study reported a statistically high significant relationship between *H. pylori* gastritis and hypertension in patients from an urban area undergoing a long-term follow-up [18]. Moreover, in a large European study involving autoptic material from over 50,000 patients, Sternby [19] observed that the obstruction of the left coronary artery was fivefold more frequently associated with prior duodenal or gastric ulcers than in those without peptic ulcer disease in subjects aged 40 to 59 years. Accordingly, a significantly higher prevalence of *H. pylori* infection has been reported in patients with coronary heart disease than among controls [20,21]. Furthermore, it has been demonstrated that *H. pylori* infection may contribute to plaque instability through anti-*H. pylori* CagA and/or VacA antibodies able to cross-react with antigens of both normal and atherosclerotic blood vessels [22,23]. In a recent meta-analysis including 40 studies for a total of 19,691 patients, *H. pylori* infection increased the risk of cardiovascular events (odds ratio [OR], 1.51), and the risk was even greater for *H. pylori* CagA positive strains (OR, 1.73). Among cardiovascular events, the risk was highest for myocardial infarction (OR, 1.80) and cerebrovascular disease (OR, 1.54) [24]. Interestingly, an additional meta-analysis showed that subjects younger than 60 years old without cardiovascular risk factors were more prone to develop atherosclerotic lesions if infected with *H. pylori* [25].

It has been hypothesized that one mechanism by which *H. pylori* infection may predispose to CVD is by increasing blood pressure values.

In a cross-sectional study performed in 5168 healthy adult Chinese subjects, it was observed that *H. pylori* infection was associated with an increased prevalence of hypertension (OR, 1.23), after adjustment for potential confounders [26]. Similar results were confirmed in different countries including Iran [27], England [28], and Japan [29], among others. More importantly, in a prospective study, *H. pylori* eradication was followed by a significant decrease in blood pressure values in hypertensive patients, especially for diastolic blood pressure [30]. These results highlight a cause-effect relationship between *H. pylori* infection and the occurrence of hypertension. However, other studies did not find evidence of increased risk after adjusting for potential confounders [31,32].

Based on these results, in this study we explore the relationship between *H. pylori* infection, blood hypertension, and the presence of atherosclerotic carotid lesions in a large cohort of patients undergoing upper endoscopy to provide further evidence about this issue.

## 2. Materials and Methods

### 2.1. Study Design

This was a retrospective, cross-sectional study aimed to evaluate the association between blood hypertension, carotid lesions, and *H. pylori* infection. Traditional risk factors for hypertension and atherosclerosis, including sex, age, excess of weight, smoking, diabetes, and dyslipidemia were considered as potential confounders.

### 2.2. Study Participants

The clinical records of adult patients who underwent upper endoscopy for any reason from January 2002 to September 2019 at the Gastroenterology Unit of the University of Sassari, Italy, were retrieved. Each patient had been evaluated by a trained gastroenterologist to collect a comprehensive medical history including a previous diagnosis of hypertension and all medications currently taken. Hypertension was defined as systolic blood pressure ≥140 mm Hg and/or a diastolic blood pressure ≥90 mm Hg in at least three measurements and matched with antihypertensive treatment. Only subjects previously assessed by a cardiologist who confirmed hypertension diagnosis were included in the analysis, while subjects for which no reliable evidence of established hypertension was available were considered as controls from the analysis.

### 2.3. Exclusion Criteria

Patients younger than 18 years were excluded from the study. Unknown *H. pylori* status and/or absence of gastric biopsies were considered additional exclusion criteria. For patients who underwent multiple EGDs within the study period, only data from the index endoscopy were recorded.

### 2.4. Helicobacter pylori Status

All patients underwent an upper endoscopy. Patients were referred to the endoscopy service by family physicians and/or specialists for a number of reasons, including dyspeptic or reflux symptoms, screening for celiac disease, presence of alarm signs and/or symptoms, varices detection, assessment of hemorrhage risk, and follow-up, among others. During the exam, a minimum of four non-targeted gastric biopsy specimens were taken, when not contraindicated, as previously described [33]. Briefly, gastric specimens were collected from the antrum, angulus, and two from the corpus. *Helicobacter pylori* infection was defined by the presence of the bacteria on histological examination. Gastric mucosa replaced by intestinal epithelium was classified as intestinal metaplasia and loss of glands as atrophy [33]. Morphology was assessed by a dedicated GI-pathologist. In the case of a chronic-active gastritis and absence of the bacteria, the infection was confirmed by the stool antigen test or ¹³C-Urea breath test (¹³C-UBT) as recommended [34].

### 2.5. Carotid Ultrasonography and Atherosclerosis Detection

Carotid ultrasonography was performed in a subgroup of 333 patients. All examinations were performed using linear-array transducers, with at least a frequency of emission of 7.5 MHz. Common carotid artery, bifurcation, and internal and external carotid arteries were bilaterally interrogated with the patient lying in the supine position. Intima-media thickness (IMT) and diameter were measured on both sides in the common carotid artery (about 1 cm proximally from carotid bifurcation) with the leading-edge method [35] and averaged. In order to reduce the confounding impact of differences in blood pressure on carotid IMT 2 mean, the carotid cross-sectional area was calculated from the average diameter and intima-media thickness as the difference between the area that encompasses the arterial diameter and the arterial luminal area [36], according to the formula:

(1) $$CCSA=\pi {\left(\frac{CA\;diameter+2\times \left(mean\;IMT\right)}{2}\right)}^{2}-\pi {\left(\frac{CA\;diameter}{2}\right)}^{2}$$

where *CCSA* is the carotid cross-sectional area, *CA* is the carotid artery, and *IMT* is the intima-media thickness.

Carotid plaques were defined as focal structures encroaching into the arterial lumen of at least 0.5 mm or 50% of the surrounding *IMT* value or demonstrating a thickness ≥ 1.5 mm as measured from the intima-lumen interface to the media-adventitia interface [35]. The atherosclerotic burden was assessed by the carotid plaque score, calculated as the number of carotid segments (common, bifurcation, internal carotid artery) bilaterally presenting atherosclerotic plaques (range 0 to 6) [37].

### 2.6. Ethical Considerations

An Institutional Review Board approval was obtained from the local ethics committee: Comitato di Bioetica, Azienda Ospedaliero-Universitaria di Sassari, Italy (Protocol code 2099/CE, 2014).

### 2.7. Statistical Analysis

Continuous variables were expressed as means ± standard deviation (SD) and compared using the Student’s *t*-test. For categorical variables, expressed as frequencies, the chi-square test was used. The BMI was calculated by using the formula weight (kg)/height (m)²; overweight and obesity were defined as a BMI of 25–29 and ≥30 kg/m², respectively. *Helicobacter pylori* status was stratified into (i) current infection (when a chronic-active gastritis was present in addition to the bacteria on gastric samples); (ii) when *H. pylori* infection with atrophy/metaplasia/dysplasia was observed it was interpreted as a long-lasting infection; and (iii) previous *H. pylori* infection, in the case of a positive clinical history of *H. pylori* infection, successfully eradicated, without evidence of the bacteria and/or chronic-active gastritis on histological specimens. The sample size of patients undergoing carotid ultrasonography examination was estimated to be at least of *n* = 141 per group required to provide 80% power to detect a 1 mm difference in the mean carotid cross-sectional area around 17 ± 3 mm with a significance of 0.05 (two-sided α). To assess the risk of hypertension and the presence of carotid lesions associated with *H. pylori* infection, multivariable logistic regression models were used by calculating the ORs and their 95% confidence interval (CI). Since hypertension was sporadic before age 30 years, in the regression models the risk of hypertension was calculated only in subjects older than the third decade both unadjusted and adjusted for age, BMI, sex, smoking status, diabetes, and dyslipidemia. ORs were calculated according to *H. pylori* status and more specifically for long-lasting and current infection.

All statistical analyses were performed using SPSS Statistics version 22.0 (Chicago, IL, USA). A two-sided *p* < 0.05 was considered to be statistically significant.

## 3. Results

### Descriptive Statistics

Data from a total of 7152 clinical records (female 4532; 63.4%) were available for the analysis (Table 1). All patients were from Northern Sardinia, sharing a similar genetic background. An established diagnosis of blood hypertension was available in 2039 (28.5%) patients. Older age, overweight and obesity, hypercholesterolemia, diabetes, and former or current smokers were significantly more frequent in patients with hypertension (Table 1). Interestingly, in patients with long-lasting *H. pylori* infection, hypertension was detected more frequently, and the difference with patients without infection was statistically significant (*p* < 0.01) (Table 1).

**Figure 1:**
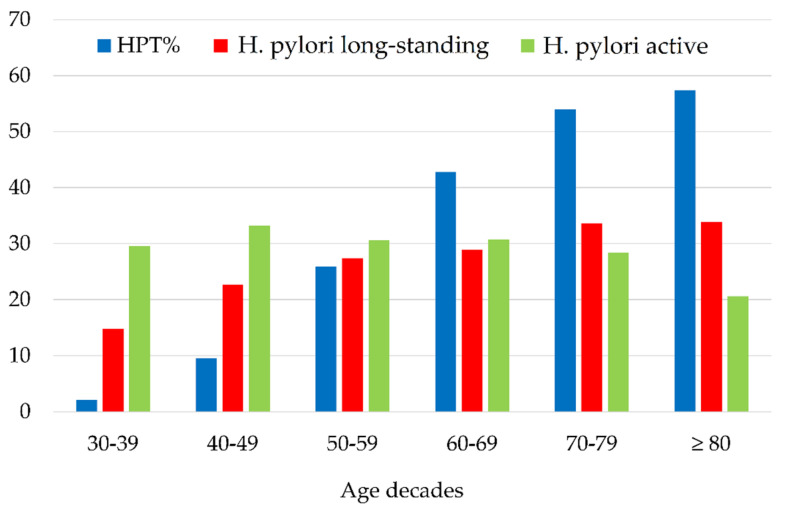
Distribution of blood hypertension and *Helicobacter pylori* infection according to age decades. HTP: hypertension.

As expected, blood pressure tended to rise in oldest decades showing a near relationship with the percentage of *H. pylori* infection represented in the graph of Figure 1.

Table 2 lists the unadjusted and adjusted ORs and their 95% CIs for all variables analyzed by the logistic regression. Long-lasting *H. pylori* infection was confirmed as a risk factor for developing blood hypertension in addition to a previous *H. pylori* infection with ORs of 1.31 and 1.17, respectively (Table 2). In contrast, a current *H. pylori* infection—characterized only by the presence of chronic-active gastritis—was not associated with hypertension. After adjusting for all study covariates, i.e., oldest age, overweight, obesity, dyslipidemia, diabetes, and cigarette smoking, long-lasting infection remained a strong risk factor (Table 2).

After adjusting for all covariates, long-lasting *H. pylori* infection remained a strong risk factor for blood hypertension in both sexes (Supplementary Materials, Table S1).

Table 3 shows carotid parameters according to *H. pylori* status in a subgroup of 333 patients undergoing carotid ultrasonography. This subgroup did not differ from the general cohort by sex but included older subjects (62.1 ± 14.2 vs. 52.0 ± 17.3, *p* < 0.0001). The overall prevalence of *H. pylori* infection was 49.8%. The analysis detected a statistically higher prevalence of right, left and any carotid plaque in patients with a long-lasting *H. pylori* infection, but not for current infection when compared to *H. pylori* negative patients (Table 3).

Results of the logistic regression analysis with the long-lasting *H. pylori* infection as exposure with hypertension and carotid atherosclerosis lesions as outcomes, adjusted for age as a continuous variable BMI, smoke, dyslipidemia, and diabetes are listed in Table 4 in the subgroup of patients undergoing carotid ultrasonography. The long-lasting *H. pylori* infection was significantly associated with the risk of developing atherosclerotic carotid lesions, independently from the traditional CVD risk factors.

## 4. Discussion

It has been reported that, beside traditional risk factors, infectious diseases may also have a role in the pathogenesis of atherosclerosis. Acute infections may increase temporarily the risk of cardiovascular events [6,7]. For example, the influenza virus and coronavirus 2 have been associated with an increased risk of acute myocardial infarction within a few days from onset in infected patients [6,7]. In addition, chronic infection may induce a systemic low-grade, persistent inflammatory process and endothelial dysfunction responsible for cardiac events. Moreover, exposure to persistent high bacterial endotoxin burden is associated with an increased risk of atherosclerosis [10].

Among infectious agents, the most studied organisms related to atherosclerosis are Chlamydia pneumoniae [9], cytomegalovirus [5] and *H. pylori* [8]. *Helicobacter pylori* is still a common infection across countries especially in less affluent populations [12]. The pathogen is etiologically related to gastritis, peptic ulcer disease and gastric cancer, although the infection has been progressively associated with several extra-GI disorders comprising blood hypertension, metabolic syndrome, atherosclerosis and, more generally, CVD [38,39].

Studies involving *H. pylori* infection in the rise of blood pressure are numerous [27,40,41,42,43]. Accordingly, in our study we found a statistically higher prevalence of blood hypertension, confirmed by the antihypertensive treatment, in patients with *H. pylori* infection, compared with patients without, after adjusting for covariates such as age, smoke, overweight/obesity, hypercholesterolemia, and diabetes, traditionally considered major risk factors for blood hypertension.

Katayoun Vahdat et al. observed that *H. pylori* seropositivity was significantly and independently associated with essential hypertension, with a OR similar to that found in our study (1.37), and the risk of hypertension was even higher in the presence of coinfection of *H. pylori* with C. pneumoniae (OR, 1.68) after adjusting for age, sex, chronic low-grade inflammation, and CVD risk factors [27]. A significantly higher frequency of seropositivity against *H. pylori* infection in patients with hypertension, in respect to controls, was also detected in England (*p* = 0.007) and Japan (*p* = 0.014), respectively [28,29]. In a cross-sectional study conducted in a large population of 37,263 healthy subjects, after controlling for confounders by multiple linear regression analysis, seropositivity for *H. pylori* was significantly and positively associated with diastolic hypertension [44]. A higher prevalence of *H. pylori* infection, detected by ¹³C-UBT, was also reported in healthy subjects with blood hypertension undergoing a routine checkup [45]. In a community-based cross-sectional study, *H. pylori* infection assessed by ¹³C-UBT showed a small effect on systolic pressure and, although it was statistically significant, the authors concluded it was not clinically relevant [43]. On the contrary, in a Czech cohort, blood pressure was not influenced by *H. pylori* infection [46].

Contrasting results among the different studies may be due by several reasons, including adjustment for potential confounders and assessment of *H. pylori* status.

*Helicobacter pylori* infection is generally acquired in childhood and causes a typical gastritis characterized by a morphological pattern of acute-on-chronic inflammation, and once established, the infection is typically lifelong if otherwise not diagnosed and treated. Long-lasting infection is characterized by replacement of parietal cells with gastric atrophy, reduction of glands and acid secretion, and eventually, a substitution with intestinal-type epithelium [47]. For this reason, it is commonly and universally accepted that histological signs of atrophy, intestinal metaplasia/dysplasia invariably denote a long-lasting *H. pylori* infection. This scenario is not inevitable and occurs in a subpopulation of infected adults at a rate of 1% to 2% per year [48]. The presence of the bacteria in the stomach enhances a serum immune response, characterized by anti–*H. pylori* IgG antibodies. However, serologic tests remain positive for a long time after *H. pylori* eradication, thus the presence of IgG in the serum is not a reliable marker to delineate between an active or past infection [34]. Instead, noninvasive tests such as ¹³C-UBT and the stool antigen test could effectively detect an active infection. Although both tests have a robust accuracy, they are unable to assess the gastric mucosa status [34]. The sequence of events leading from *H. pylori* gastritis to gastric premalignant lesions (e.g., atrophy, metaplasia, and/or dysplasia) is a long process associated with a systemic, low-grade proinflammatory state. In the majority of studies analyzing the association between *H. pylori* infection and blood hypertension, the *H. pylori* status was tested by serology or ¹³C-UBT, making it possible for the two tests to bias the results: serology could have captured several subjects already eradicated, and the ¹³C-UBT does not discriminate between patients with a long-lasting (associated with a persistent inflammatory state) or recent infection. In order to minimize these problems in our study, for the first time we stratified patients into those having a previous, successfully treated *H. pylori* infection, those having a current infection (chronic active gastritis), and those having a long-lasting infection, based on the presence of metaplasia/atrophy/dysplasia on histological examination. Consistently, in patients harboring the infection for long time, the association with blood hypertension and atherosclerotic carotid lesions, including plaques, was statistically significant whereas in the group displaying current active infection was not, after adjusting for covariates in both sexes.

### Limitations

This study has a number of limitations that need to be mentioned. First, this was a retrospective cross-sectional study not suitable to assess a causal effect. Moreover, in retrospective studies there is a poor control over the exposure factor. However, in our study *H. pylori* status was defined by histology on gastric specimens and the duration of gastritis by the type of gastric lesions detected on morphological examination. In addition, in the absence of the bacteria in gastric specimens strongly suspected for *H. pylori* gastritis, the infection was further verified by ¹³C-UBT or stool antigen test. For these reasons, we are confident that in our study very few *H. pylori* positive patients have been missed from the analysis. Second, although we controlled for the most known risk factors for blood hypertension through the logistic regression model, additional risk factors such as markers of systemic inflammation, familiarity, genetics, diet, and especially salt intake, were not available. Third, a number of subjects with an unknown blood hypertension may have been lost from the group of *H. pylori* positive or negative patients with a consequent underestimation of the population with blood hypertension. However, we can assume that the effect was minimal and affected both subgroups in a similar fashion. Finally, only a small number of patients undergoing upper endoscopy underwent a carotid ultrasound examination, potentially decreasing the generalizability of our findings to the entire population.

## 5. Conclusions

In summary, according to the majority of studies on this topic, our result suggests that *H. pylori* infection, especially the long-lasting infection, is an independent risk factor for blood hypertension and for the presence of carotid plaques after adjusting for potential confounders. However, the results obtained in our study should be critically evaluated given its retro-spective design, and should be further validated in additional prospective studies before claiming a cause-effect relationship. Confirmation of *H. pylori* infection as an additional CV risk factor may allow to better stratify patient risk, and the bacteria eradication an ad-ditional tool in the management of blood hypertension.

## Figures and Tables

**Table 1:** Descriptive statistics in 7152 study participants according to blood hypertension.

Variables	No Hypertension (*n* = 5113)	Hypertension (*n* = 2039)
Sex, *n* (%)		
Male	1891 (37.0)	729 (35.8)
Female	3222 (63.0)	1310 (64.2)
Age, *n* (%)		
30–39	1165 (22.8)	26 (1.3)
40–49	1231 (24.1)	142 (7.0) **
50–59	1119 (21.9)	392 (19.2) **
60–69	932 (18.2)	699 (34.3) **
70–79	525 (10.3)	613 (30.1) **
≥80	141 (2.8)	167 (8.2) **
Body mass index, *n* (%)		
<25 kg/m²	2973 (58.1)	874 (42.9)
25–29 kg/m²	1583 (31.0)	804 (39.4) **
≥30 kg/m²	557 (10.9)	361 (17.7) **
Smoke, *n* (%)		
Never smoker	2683 (52.5)	1094 (53.7)
Former smoker	218 (4.3)	111 (5.4) *
Current smoker	2212 (43.3)	834 (40.9)
Dyslipidemia, *n* (%)		
No	4758 (93.1)	1576 (77.3)
Yes	355 (6.9)	463 (22.7) **
Diabetes, *n* (%)		
No	4871 (95.3)	1649 (80.9)
Yes	242 (4.7)	390 (19.1) **
History of *H. pylori* infection ^#^, *n* (%)		
No	4972 (97.2)	1977 (97.0)
Yes	141 (2.8)	62 (3.0)
*H. pylori* status, *n* (%)		
No infection	2296 (44.9)	837 (41.0)
Long-lasting *H. pylori* infection ^§^	1254 (24.5)	607 (29.8) **
Current *H. pylori* infection ^‡^	1563 (30.6)	595 (29.2)

* *p* < 0.05; ** *p* < 0.01; ^#^ A positive clinical history of *H. pylori* infection, successfully eradicated, without evidence of the bacteria and/or chronic-active gastritis on histological specimens. ^§^ Presence of *H. pylori* infection associated with atrophy/metaplasia/dysplasia was interpreted as a long-lasting infection. ^‡^ Presence of chronic-active gastritis in addition to the bacteria on gastric specimens.

**Table 2:** Logistic regression analysis for blood hypertension in 7152 study participants.

Variables	Unadjusted OR (95% CI)	Adjusted OR (95% CI)
Sex		
Male	Ref.	Ref.
Female	1.05 (0.95–1.17)	1.13 (1.00–1.28) *
Age		
30–49 years	Ref.	Ref.
50–59 years	4.99 (4.11–6.07) **	4.36 (3.58–5.31) **
60–69 years	10.70 (8.89–12.86) **	8.54 (7.07–10.31) **
70–79 years	16.65 (13.70–20.24) **	12.98 (10.62–15.86) **
≥80 years	16.89 (12.85–22.20) **	13.48 (10.17–17.87) **
Body mass index		
<25 kg/m²	Ref.	Ref.
25–29 kg/m²	1.72 (1.54–1.93) **	1.33 (1.17–1.52) **
≥30 kg/m²	2.20 (1.89–2.57) **	1.92 (1.61–2.28) **
Smoke		
Never smoker	Ref.	Ref.
Former smoker	0.92 (0.83–1.03)	0.98 (0.75–1.28)
Current smoker	1.25 (1.02–1.59) *	1.13 (0.98–1.94)
Dyslipidemia		
No	Ref.	Ref.
Yes	3.94 (3.39–4.57) **	2.38 (2.02–2.81) **
Diabetes		
No	Ref.	Ref.
Yes	4.76 (4.02–5.64) **	3.94 (2.17–4.36) **
History of *H. pylori* infection		
No	Ref.	Ref.
Yes	1.19 (1.07–1.32) *	1.13 (1.01–1.26) *
*H. pylori* status		
No infection	Ref.	Ref.
Long-lasting infection ^§^	1.31 (1.16–1.46) **	1.17 (1.02–1.35) *
Current infection ^‡^	0.93 (0.84–1.05)	0.99 (0.86–1.14)

* *p* < 0.05; ** *p* < 0.01, ^§^ Presence of *H. pylori* infection associated with atrophy/metaplasia/dysplasia was interpreted as a long-lasting infection. ^‡^ Presence of chronic-active gastritis in addition to the bacteria on gastric specimens.

**Table 3:** Mean and standard deviation of carotid parameters in 333 study participants according to the long-lasting (*H. pylori* with atrophy/metaplasia/dysplasia), and current *H. pylori* (Hp) infection.

Variables	Long-Lasting Hp Infection (*n* = 91)	Current Hp Infection (*n* = 75)	Hp Negative (*n* = 167)
Mean carotid cross-sectional area (mm²)	17.4 ± 5.6 *	15.1 ± 4.5	16.1 ± 5.1
Mean Intima-media thickness (mm)	0.77 ± 0.16 *	0.72 ± 0.14	0.73 ± 0.14
Right carotid plaques			
No	33 (36.2)	79 (52.0)	90 (53.9)
Yes	58 (63.8) *	36 (48.0)	77 (46.1)
Left carotid plaques			
No	39 (42.9)	43 (57.3)	98 (58.7)
Yes	52 (57.1) *	32 (42.7)	69 (41.3)
Any carotid plaque			
0	24 (26.4)	35 (46.7)	79 (47.3)
1	23 (25.3) *	15 (20.0)	32 (19.2)
2	44 (48.4) **	25 (33.3)	56 (33.5)

* *p* < 0.05; ** *p* < 0.01.

**Table 4:** Logistic regression analysis with *H. pylori* infection as exposure and carotid parameters as the outcome.

Variables	Patients with Carotid Parameters (*n* = 333)	
	**Long-Standing** ***H. pylori*** **Infection**	**Current** ***H. pylori*** **Infection**
Hypertension ^§^	1.85 (1.04–3.30) *	1.05 (0.57–1.90)
Carotid cross-sectional area (>90%) ^#^	1.51 (0.79–2.88)	1.45 (0.75–2.81)
Right carotid plaque ^#^	1.70 (0.92–3.14)	1.25 (0.66–2.35)
Left carotid plaque ^#^	1.69 (0.90–3.13)	1.81 (0.95–3.45)
Any carotid plaque ^#^	2.15 (1.14–4.09) *	1.52 (0.81–2.85)

* *p* < 0.05; ^§^ based on the cardiologist diagnosis and current use of anti-hypertensive medications. ^#^ adjusted for age as a continuous variable, sex, body mass index, cigarette smoke, dyslipidemia, and diabetes.

## Data Availability

The data presented in this study are available on request from the corresponding author.

## Supplementary Materials

The following are available online at https://www.mdpi.com/article/10.3390/jcm11092282/s1, Table S1: Logistic regression for blood hypertension in male and female participants.
