# Supplementary material for: Increased Risk to Develop Hypertension and Carotid Plaques in Patients with Long-Lasting Helicobacter pylori Gastritis
Source: J Clin Med. 2022 Apr 19;11(9):2282. doi: 10.3390/jcm11092282 (PMC9104887; doi:10.3390/jcm11092282)
Supplement: Supplementary file 1 [file jcm-11-02282-s001.zip › jcm-1631434-supplementary.pdf]

Table S1. Logistic regression for blood hypertension in male and female participants.

Variables	Males		Females	
	**Unadjusted** OR (95% CI)	**Adjusted** OR (95% CI)	**Unadjusted** OR (95% CI)	**Adjusted** OR (95% CI)
Age (years)				
30-49 years	Ref.	Ref.	Ref.	Ref.
50-59 years	4.78 (3.47 – 6.59) **	3.92 (2.82 – 5.44) **	5.12 (4.02 – 6.54) **	4.62 (3.60 – 5.91) **
60-69 years	8.94 (6.60 – 12.11) **	6.97 (5.11 – 9.52) **	11.87 (9.40 – 14.98) **	9.68 (7.62 – 12.29) **
70-79 years	14.41 (10.51 – 19.77) **	10.89 (7.87 – 15.07) **	18.23 (14.22 – 23.36) **	14.53 (11.25 – 18.75) **
≥ 80 years	11.28 (7.27 – 17.52) **	8.66 (5.49 – 13.65) **	20.54 (14.45 – 29.19) **	16.86 (11.73 – 24.23) **
Body mass index				
< 25 kg/m²	Ref.	Ref.	Ref.	Ref.
25-29 kg/m²	1.48 (1.22 – 1.78) **	1.17§ (0.95 – 1.44)	1.92 (1.66 – 2.22) **	1.44§ (1.22 – 1.69) **
≥ 30 kg/m²	1.95 (1.51 – 2.51) **	1.43§ (1.07 – 1.91) *	2.37 (1.96 – 2.86) **	2.26§ (1.81 – 2.81) **
Smoke				
Never smoker	Ref.	Ref.	Ref.	Ref.
Former smoker	1.04 (0.87 – 1.24)	0.99§ (0.82 – 1.21)	0.87 (0.77 – 1.01)	0.88§ (0.75 – 1.02)
Current smoker	1.92 (1.41 – 2.63) **	1.66§ (1.19 – 2.33) **	0.93 (0.80 – 1.07)	0.79§ (0.52 – 1.21)
Dyslipidemia				
No	Ref.	Ref.	Ref.	Ref.
Yes	4.60 (3.47 – 6.11) **	2.92§ (2.15 – 3.97) **	3.71 (3.11 – 4.43) **	2.18§ (1.79 – 2.65) **
Diabetes				
No	Ref.	Ref.	Ref.	Ref.
Yes	4.81 (3.73 – 6.21) **	2.37§ (1.80 – 3.12) **	4.83 (3.84 – 6.07) **	2.10§ (1.64 – 2.69) **
History of *H. pylori* infection				
No	Ref.	Ref.	Ref.	Ref.
Yes	1.19 (0.67 – 2.09)	1.37# (0.73 – 2.56)	1.22 (0.86 – 1.73)	1.02# (0.67 – 1.55)
H. pylori status				
No infection	Ref.	Ref.	Ref.	Ref.
Long-lasting infection§	1.34 (1.09 – 1.65) *	1.27 (1.01 – 1.60) *	1.33 (1.13 – 1.55) **	1.25 (1.06 – 1.48) **
Current infection‡	0.95 (0.77 – 1.12)	0.97# (0.77 – 1.17)	1.09 (0.94 – 1.27)	1.08# (0.92 – 1.26)

§ adjusted for all covariates except for Helicobacter pylori infection; # adjusted for sex, age groups, BMI, smoke, dyslipidemia and diabetes.
* p <0.05; ** p < 0.01.
